# First Report of 3-Oxothiolase Deficiency in Iran

**DOI:** 10.5812/ijem.10960

**Published:** 2014-04-01

**Authors:** Kobra Shiasi Arani, Babak Soltani

**Affiliations:** 1 Research Center for Biochemistry and Nutrition in Metabolic Disorders, Kashan University of Medical Sciences, Kashan, IR Iran

**Keywords:** Methylacetoacetyl-CoA Thiolase Deficiency, Beta-ketothiolase Deficiency, 3-Oxothiolase Deficiency, Acidosis

## Abstract

**Introduction:**

Mitochondrial acetoacetyl-CoA thiolase (3-oxothiolase) deficiency is a rare metabolic disorder involving ketone body metabolism characterized by acute attacks of vomiting, acidosis, ketosis, and lethargy along with some laboratory criteria including excessive excretion of 2-methyl-3-hydroxybutyric acid in urine.

**Case Presentation:**

This is a case report of 3-oxothiolase deficiency in a young Iranian boy with presentation of intractable vomiting and severe metabolic acidosis following a common cold in six months of age with abundant urinary 2-methyl-3- hydroxybutyric acid.

**Discussion:**

This is the first Iranian 3-oxothiolase deficiency case report as searched in the literature. Because of the high rate of consanguineous marriages in Iran, physicians should consider the 3-oxothiolase deficiency in the differential diagnosis of any patient with intractable vomiting and severe metabolic acidosis.

## 1. Introduction

The 3-oxothiolase deficiency is a rare disorder involving ketone body metabolism. The enzymatic defect is the deficiency of mitochondrial acetoacetyl CoA thiolase and the responsible gene is on chromosome 11q22.3-q23.1 (1-3) with autosomal recessive inheritance. There is considerable heterogeneity in clinical features, while a unique presentation is the attacks of massive ketosis and acidosis. Rarely, patients have neonatal onset; however, most of them begin first in late infancy or childhood. The attacks are usually triggered by infections and other causes of increased metabolism. Dehydration, lethargy, hyperventilation, coma, and death might occur during these episodes. Noticeable patients have a history of sibling deaths in early years of age (4, 5).

The frequency of attacks decreases with age. Other features are hyperglycemia, vomiting, seizures, mental retardation, central hypotonia, ataxia, and speech problems (4). Specific organic aciduria is the diagnostic key of this disorder. The main metabolites are 2-methyl- 3-hydroxybutyric acid, tiglylglycine, and 2-methylacetoacetic acid. They are excreted constantly in patient’s urine and have trace levels in normal urine (4, 5). There is no definite treatment for the disorder. According to considerable heterogeneity in clinical presentations, the treatment schedules are individualized. During fever or gastrointestinal disturbance, prolonged fasting is hazardous and intravenous glucose is beneficial and may prevent the ketoacidotic crisis. Large volume of water and electrolytes are prudent during the furious acidotic episode. Intravenous carnitine administration during attacks followed by long-term orally intake to esterify and remove tiglyl CoA and the other accumulated CoA esters are practical. It is appropriate to limit the use of isoleucine in some patients (6).

## 2. Case Presentation

A 6-month-old boy presented with intractable vomiting and a history of three-day symptoms of common cold. On examination, he was conscious, febrile, tachypneic, dehydrated, and hypotonic with an acetone-like breath odor. The patient was the first child of consanguineous parents. There was no family history of similar disease. He had normal growth and development prior to the recent disorder. Initial laboratory studies showed severe metabolic acidosis (PH: 7.06, bicarbonate: 8.5) and severe ketonuria. Blood glucose, electrolytes, liver enzymes, and thyroid function tests had normal results. Routine sepsis workup and CSF analysis revealed negative results for infections. The patient had high level of plasma lactate (27 and 35 mmol/L in two separate occasions) and normal level of blood ammonia. Urine organic acids analysis by gas chromatography/mass spectrometry (GC/MS) indicated a very high level of 2-methyl-3-hydroxybutyric acid (MHB) (1335 mmol/mol creatinine) and trace amount of tiglylglycine (TG). Chromatography of plasma amino acids with HPLC method showed a slight increase of serine, glutamine, and aspartate amino acids. Brain MRI at the seventh month of life showed mild hypomyelination of white matter and the results were normal for other parameters. Fluid and alkaline therapy were initiated in the acute phase of disease and L-carnitine and low isoleucine diet followed to treat the patient. The patient developed regression in developmental milestones after the first attack of his disease. The child was able to sit prior of the disease but at 16 months of age he became able to sit again. The walking with assistance started at 20 months of age. The patient had developed recurrent abnormal movements of tongue, salivary drooling, and dystonia in upper and lower extremities at seven months of age. Dystonia relatively responded to levodopa and trihexyphenidyl; however, it recurred by discontinuation of drugs.

## 3. Discussion

The 3-oxothiolase deficiency is a rare disorder and less than 100 cases are reported by now (2). There is no previous report of this disorder in Iran. Our patient was a typical case of the disease. These patients are marked by recurrent attacks of severe ketosis mostly caused by ketolysis defects, the main probable mechanism of excessive production of acetoacetate (7). There is significant variety in reported cases, but the specific characteristics are the episodes of massive ketosis and acidosis. Sometimes attacks are marked by vomiting and are occasionally accompanied by dehydration, lethargy, hyperventilation, and coma (4, 8). The disease is usually detected in late infancy or childhood. Our patient had 6 months of age at presentation. Infections are the most common triggering causes of attacks; n our patient, the common cold was the triggering factor. Ketoacidotic attacks are the most common clinical manifestations. Intelligence may be normal occasionally but in others, developmental delay or speech disorders exist (9-11). Although our patient developed regression in developmental milestones after the first attack of his disease, he recovered partially with time. Severe neurologic delay was reported recently in four cases and the development was slow before the initial acidotic episodes in all of them. Severe central hypotonia was reported in all patients (12). Our patient also had hypotonia. Ataxia was reported in some patients and severe headaches in the others (4, 13, 14). In some patients, brain MRI showed high intensity T2 lesions in the posterolateral putamen, but this pattern is not diagnostic (12). Recently, O'Neill et al reported a five-year-old girl with Betha-ketothiolase deficiency with isolated focal T2 hyperintensities involving the globi pallidi (15). A 19-month-old patient had cognitive and motor development delay, spastic diplegia, dysmorphism, and occipital periventricular white matter lesions on MRI scan of the brain (16). Brain MRI of our patient showed mild hypomyelination of white matter and had normal findings in other parameters. Congestive cardiac myopathy and seizure have been reported (17), but cardiac function was normal in our patient and he had not seizure. 

The number of acute attacks decreases by age and the latest reported episode was at 10 years of age. In a report of 26 patients, three patients had no episodes, 11 patients had only one, and 12 patients had recurrent attacks of ketoacidosis (4). Fukao et al. reported one patient that experienced two ketoacidotic episodes at the age of 9 months and 3 years, and no further episodes until the age of 25 years. She had normal growth and development and two uncomplicated pregnancies (3). He also reported two patients that were identical twins who presented their first ketoacidotic crisis simultaneously at the age of 3 years and 4 months (3).

Organic aciduria is diagnostic for this disorder. The main metabolites are 2-Methyl-3-hydroxybutyric acid, tiglylglycine, and 2-methylacetoacetic acid. These are excreted constantly in the patient’s urine and are detected in very low levels in normal urine samples. Organic acid analysis is available and can provide the diagnosis as in our patient. Elevated blood ammonia and lactate are unusual; our patient had high level of plasma lactate and normal level of blood ammonia. 

Urine amino acid levels are usually normal, but three patients with hyperglycinuria were reported (11, 14, 18). Generally, the levels of 2-methyl-3-hydroxybutyric acid are significantly higher than 2-methylacetoacetic acid, and sometimes the later might be undetectable. The 2-Methyl-3-hydroxybutyric acid might be detected in urine in levels of 200 to 1000 mmol/mol of creatinine under healthy conditions, increasing in acute illness or following use of protein or isoleucine to as high as 14400 mmol/mol creatinine (9, 14). Healthy individuals excrete it in levels lower than 10 mmol/mol creatinine. Tiglylglycine is detected in levels up to 7000 mmol/mol creatinine; however, some patients do not excrete tiglylglycine (17, 19). In acute ketosis, key metabolites can be confusing. Furthermore, severe ketosis may cause excretion of 3-hydroxy acids, including 2- methyl-3-hydroxy-butryric acid in quantities as high as 200 mmol/mol creatinine and also 3-hydroxyisovaleric acid (17). In cases of propionic acidemia, 2-methyl-3-hydroxybutryic acid and 2-methylacetoacetic acid can also be detected in the urine during ketosis (20). The diagnosis of patients with propionic acidemia can be properly made by the excretion of 3-hydroxypropionic acid and methylcitric acid. In our patient, 3-hydroxyisovaleric acid, methylcitric acid and 3-hydroxypropionic acid metabolites were absent in urine assay.

3-oxothiolase deficiency is a rare metabolic disorder involving ketone body metabolism. Because of the high rate of consanguineous marriages in Iran, physicians should consider the 3-oxothiolase deficiency in the differential diagnosis of any patient with intractable vomiting and severe metabolic acidosis.
